# Field notes: Children as WASH ambassadors—Insights from Pakistan’s trachoma elimination programme

**DOI:** 10.1371/journal.pntd.0013769

**Published:** 2025-12-12

**Authors:** Asad Aslam Khan, Sajjad Ali Surhio, Babar Qureshi, Zahid Awan, Ismat Zehra Juma, Opeoluwa Oguntoye, Girija Sankar, Juliana Amanyi-Enegela, Muhammad Moin, Shahbaz Ali

**Affiliations:** 1 King Edward Medical University, Lahore, Pakistan; 2 Mayo Hospital, Lahore, Pakistan; 3 Department of Ophthalmology, Liaquat University of Medical and Health Sciences, Jamshoro, Hyderabad,; 4 Christian Blind Mission, Bensheim, Germany; 5 College of Ophthalmology and Allied Vision Sciences, Lahore, Pakistan; 6 Sindh Institute of Ophthalmology and Visual Sciences, Hyderabad, Sindh, Pakistan; RTI International, TANZANIA, UNITED REPUBLIC OF

## Introduction and background

Trachoma is the leading infectious cause of blindness globally, affecting about 1.9 million people and placing over 100 million at risk [1]. This preventable disease results from repeated bacterial infections of the eye caused by *Chlamydia trachomatis*. Children between the ages of 1–9 years are particularly susceptible due to frequent nasal and ocular discharge, making them key carriers of infection within their households and communities with the disease reaching peak at around 3–6 years [2–4]. Trachoma thrives in conditions of poverty, overcrowding, limited water access, and poor sanitation and hygiene practices [2].

The World Health Organization’s recommended strategy to eliminate trachoma, known as SAFE, comprises Surgery, Antibiotics, Facial cleanliness, and Environmental improvement [1]. However, global research indicates that lasting elimination requires sustained behavioral changes, particularly in hygiene and environmental sanitation [5,6].

In Pakistan, about 1.4 million people lived in areas needing interventions for trachoma elimination as of 2022 [1]. Among these, the district of Kambar-Shahdadkot in Sindh faced significant trachoma burdens, compounded by weak sanitation infrastructure and water scarcity. The Pakistan Trachoma Elimination Project (PTEP), implemented by the Sindh Institute of Ophthalmology & Visual Sciences (SIOVS) with support from Christian Blind Mission (CBM), adopted an innovative approach engaging schoolchildren as WASH (Water, Sanitation, and Hygiene) ambassadors to promote sustainable hygiene practices within their communities. This involved facilitating the formation of about 400 WASH clubs and training the members on key practices such as hand washing, face washing, and sanitation which lead to change behavior and as such help prevent spread of trachoma [7]. Additionally, the project included construction of over 143 wash facilities, latrines inclusive wash points, and rehabilitation boreholes. This aligns with the F &E component of the SAFE strategy recommended by WHO for the elimination of trachoma.

This field reflection explores the experiences and lessons learned from involving children as active agents of change in trachoma elimination.

## Key project achievements

The project activities included formation of school WASH clubs across four hundred schools, construction of accessible WASH facilities, and training of members of school WASH clubs to facilitate adoption of hygiene practices and triggering improved hygiene behavioral change. The training focused on simple but impactful hygiene practices such as regular face and hand washing to interrupt trachoma transmission. Through creative and interactive methods, such as songs, games, and storytelling, children enthusiastically internalized key messages and became passionate advocates for cleanliness at home and school.

**Fig 1:**
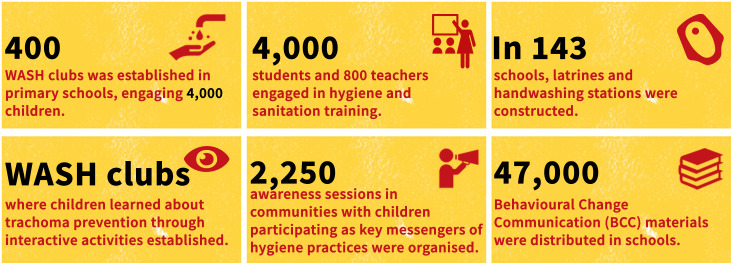
PTEP key achievements.

Fig 1 below is a summary of the key project achievements.

## Field experiences: Children as catalysts for change

The project employed a mixed-methods participatory evaluation approach, incorporating qualitative and quantitative data collection methods such as document reviews, key informant interviews (KIIs), and focus group discussions (FGDs). FGDs with students, teachers, and health workers revealed substantial improvements in hygiene behaviors.

Findings from the participatory evaluation suggest that students have enhanced awareness of trachoma transmission and enthusiastically adopted regular face-washing routines. During focus group discussions, students vividly described how they encouraged their peers and family members to adopt better hygiene habits. One WASH club member shared, *“I remind my siblings and even my parents every day about washing their faces, especially in the morning, because clean faces stop eye infections.”* This anecdote highlights the powerful influence children can have within their families, aligning with global evidence that children-led initiatives effectively promote health behaviors at household and community levels [8].

Teachers echoed these sentiments. A primary school teacher from the project reflected, *“Students quickly took ownership of hygiene practices. They started reminding each other daily, creating a culture of cleanliness without constant adult supervision.”* This peer-to-peer reinforcement not only facilitated behavior change among students but also resonated within their homes, extending the project’s reach beyond school boundaries.

Community health workers further validated the children’s role. One Lady Health Worker remarked, *“The children simplified my job. When I visited households, families were already familiar with hygiene messages because their children had effectively shared them.”* Additionally, one WASH Club captain, a student, quoted how she continued her learnt habits at home and spread the message “*Whenever I noticed someone at home not washing their hands before eating or neglecting to wash their face, I made sure to share this important message with them.”* Clearly, involving children transformed passive learning into active advocacy, significantly contributing to improved community hygiene practices.

Furthermore, KIIs with education and health officials further supported these findings. One education official commented, “*Since the children were trained, we noticed not only improved hygiene but also better school attendance due to the interest in participation in the school WASH club activities.”*

A parent of a WASH club member stated that *“It was interesting for me to observe that people are now using small designated rooms for calls of nature. I found this approach to be respectful, safe, and hygienic. I truly believe that such practices will contribute to better health and hygiene for all of us and our children, fostering a cleaner and healthier living environment.”*

Another community/education stakeholder mentioned that “*Learning about handwashing and the importance of establishing wash stations in schools has truly inspired us to adopt these practices in our daily lives. I have constructed latrines and wash stations at my home, and I am now planning to build a latrine outside my house for public use. It’s truly amazing to experience the positive changes that come from these simple yet impactful actions.*”

Such observations underline the broader benefits of school-based hygiene programmes, reinforcing global research on WASH interventions improving both health and educational outcomes [9–11].

## Reflections on implementation

Effectively implementing school hygiene education required balancing educational activities with adequate WASH infrastructure; what public health practitioners describe as integrating “soft” and “hard” components of WASH interventions [12,13]. Field experience proves that educational efforts alone were insufficient; reliable infrastructure, including functional water points and latrines, was critical for sustained hygiene practices [14].

Schools with adequate water access saw consistent hygiene behaviors, while those experiencing water shortages faced significant barriers. As a teacher explained, “*Children want to practice hygiene, but without water, our efforts cannot last.”* This underscores the necessity of reliable infrastructure as foundational to behavioral sustainability. Moreover, cross-sector collaboration involving education, health, and public works was instrumental in the project’s effectiveness. Joint efforts ensured coherent messaging, timely provision of facilities, and streamlined implementation processes.

## Key lessons and recommendations

Several key lessons emerged from the project:

- **Children as Effective Health Advocates:** Children rapidly absorb and disseminate hygiene messages within families and communities. Future interventions should consider empowering children as active promoters of WASH education.
- **Integration of Education and Infrastructure:** Reliable hygiene infrastructure is essential to enable and sustain behavior change. Educational efforts must be matched with adequate facilities to achieve lasting impacts.
- **Continuous Community Engagement:** Sustained behavioral change, especially among adults, requires ongoing dialogue and engagement. Leveraging influential community networks, including religious leaders and health workers, significantly reinforces community-wide adoption of hygiene practices.
- **Long-term Perspective:** Immediate disease prevalence reductions should not be the sole indicator of success. Cultivating long-term sustained behaviors offers significant public health benefits, reinforcing hygiene practices for future generations.
- **Institutionalizing Hygiene Education:** Incorporating hygiene practices into school curricula ensures long-term sustainability. Local education systems adopting hygiene education permanently can significantly amplify and sustain the impacts.

## Conclusion

Pakistan’s Trachoma Elimination Project demonstrates children’s capacity as powerful agents for community-wide health improvement. Positioning children as WASH ambassadors was key to successfully enhancing hygiene behaviors. Provision of WASH facilities and continuous community engagement facilitates sustained behavioral change. The combined approach of engaging education, infrastructure improvement, and continuous community dialogue demonstrated promising outcomes.

Investing in children’s WASH education is essentially investing in future community health. As a local teacher thoughtfully expressed, *“Teaching our children good hygiene today ensures healthier communities tomorrow.”* Through active child participation, communities previously burdened by trachoma have adopted sustainable hygiene practices, underscoring the potential of similar initiatives globally. The children, once merely beneficiaries, became passionate advocates, guiding their communities toward lasting health and a trachoma-free future.

Sustaining F&E would ensure that the elimination status is maintained without recrudescence. This can be corroborated with results of the Trachoma Impact Survey, which saw a reduction in prevalence of trachoma from 9.88% for TF to below the 5% threshold and the prevalence of 1.57% for TT to below the 0.2% threshold. Leading to the WHO consequently certifying Pakistan to have eliminated trachoma as a public health problem in 2024.
